# Post-synthesis from Lewis acid–base interaction: an alternative way to generate light and harvest triplet excitons

**DOI:** 10.3762/bjoc.18.83

**Published:** 2022-07-12

**Authors:** Hengjia Liu, Guohua Xie

**Affiliations:** 1 Sauvage Center for Molecular Sciences, Hubei Key Lab on Organic and Polymeric Optoelectronic Materials, Department of Chemistry, Wuhan University, Wuhan 430072, People’s Republic of China; 2 Key Laboratory for preparation and Application of Ordered Structural Materials of Guangdong Province, Shantou University, Shantou 518060, People’s Republic of China; 3 Wuhan National Laboratory for Optoelectronics, Huazhong University of Science and Technology, Wuhan 430074, People’s Republic of China

**Keywords:** excitons, fluorescence, Lewis acid, Lewis base, post-synthesis

## Abstract

The changes in absorption and emission of fluorescent materials with the introduction of Lewis acids have been frequently observed due to either physical or chemical interactions. In this mini-review, we elaborate how Lewis acids adjust the optical properties and the bandgap of luminescent materials by simple coordination reactions. It is common that fluorescent materials containing Lewis basic nitrogen heterocycles are more likely to provide the feasible band gap modulation. The essence of such phenomenon originates from Lewis acid–base coordination and adducts, which highly depends on the electron-accepting property of the Lewis acids. This intermolecular mechanism, considered as post-synthesis of new luminescent compounds offers promising applications in sensing and electroluminescence by manipulating the frontier molecular orbital energy levels of organic conjugated materials, simply based on Lewis acid–base chemistry.

## Introduction

Organic light emitting diodes (OLEDs) show great potential to dominate the next generation of flat-panel displays and efficient light sources attributed to the advantages of self-illumination, high efficiency, wide color gamut, and flexibility [1–3]. In OLEDs photons are mainly generated by radiative recombination in the emitting layer [4]. Therefore, the development of efficient luminescent materials and the exploration of new luminescent mechanisms are one of the core tasks in academic research. The most common luminescent materials are fluorescent compounds. Based on the spin statistics, the fluorescent emitters can only use singlet excitons for light generation [5]. In contrast, phosphorescent materials based on metal complexes could achieve a high internal quantum efficiency (IQE) up to 100% through intersystem crossing (ISC) [6–7]. In 2012, Adachi et al. first reported purely organic thermally activated delayed fluorescent (TADF) materials, which achieved nearly 100% exciton utilization via reverse intersystem crossing (RISC) [8]. Meanwhile, novel materials based on new luminescence mechanisms such as hybridized local and charge-transfer (HLCT) and doublet emission have been designed and demonstrated [9–10]. However, the development of these materials often requires complicated molecular design and synthesis [11–12]. Alternatively, it is also possible to produce light emission by molecular exciplexes composed of multiple molecules [13]. The exciplex contains new excited states through charge transfer between a donor molecule and an acceptor molecule. This provides a simple way to create new luminescence processes through the intermolecular interactions of existing molecules [14].

It has been reported that new emitters can be realized by adding a Lewis acid to a fluorescent conjugated compound [15–16]. Lewis acids are common complexing agents [17] and are frequently used to dope conjugated polymers to enhance their conductivity while the luminescence is completely quenched [18–19]. In contrast, in the presence of nitrogen-containing heterocycles in the fluorescent materials, the addition of a Lewis acid tended to induce red-shifted absorption and emission, shedding light on the fact that the Lewis acid interacts easily with the nitrogen-containing fluorescent materials. This interaction mechanism is the coordination between Lewis acids and bases, which can finely adjust the optoelectronic properties of the fluorescent molecules, such as band gaps, peak wavelengths, and even frontier molecular orbitals if bound together [20]. The traditional way to manipulate the optoelectronic properties of the emitters highly depends on the molecular design and structures, including linkers, donor and acceptor units, which requires complex and time-consuming molecular synthesis and optimization [21–23]. In contrast, the introduction of specific Lewis acid–base pairs in existing molecules can be utilized to achieve brand new luminescent properties. In this mini-review, we summarize unique electron donor and acceptor materials which regulate luminescent properties via Lewis acid–base interactions and briefly explain the exploration of their chemical nature and interaction mechanisms.

## Review

### Lewis acids as electron acceptors

**Figure 1:**
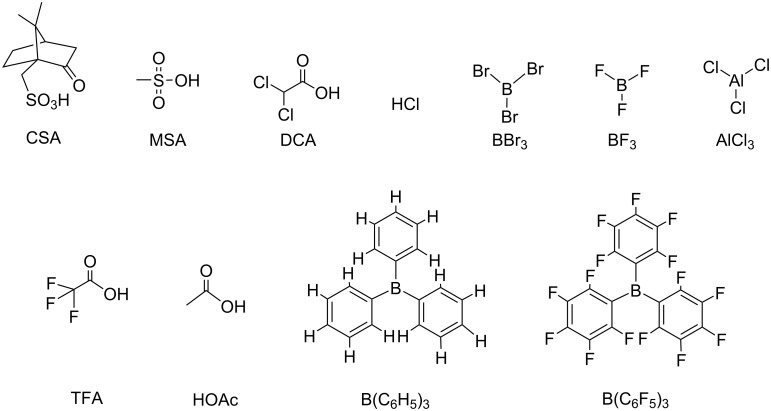
Chemical structures of Lewis acid examples.

Some Lewis acids have good solubility in common organic solvents, which makes it easy to fabricate films for optoelectronic applications [24]. Because of their strong electrophilicity [25], Lewis acids may dominate charge distributions of the fluorescent materials featured with electron-rich nitrogen-containing heterocycles, resulting in the change of energy levels and spectra. The following will illustrate Lewis acids used in the exploration of luminescent materials and mechanisms due to Lewis acid–base interactions. The chemical structures of some candidate Lewis acids are shown in Figure 1.

**Figure 2:**
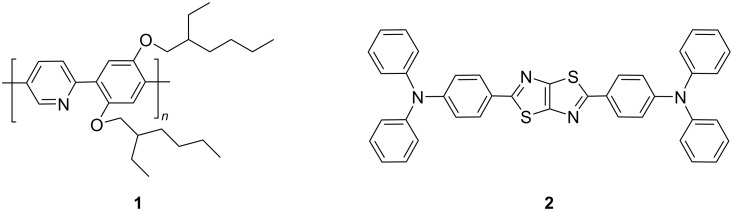
Chemical structures of Lewis basic fluorescent polymer poly{2,5-pyridylene-*co*-1,4-[2,5-bis(2-ethylhexyloxy)]phenylene} **1** and D–A–D compound 2,5-bis((*N*,*N*-diphenylamino)phenyl)thiazolothiazole **2**.

**Figure 3:**
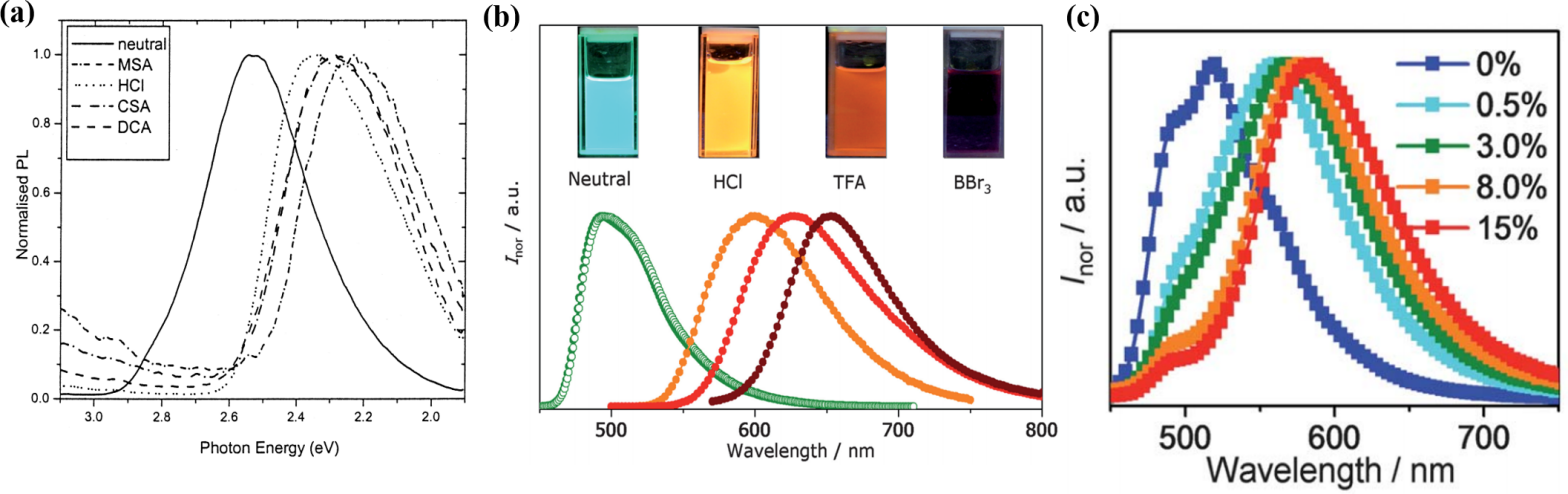
(a) Normalized PL spectra of films with compound **1** doped with different Lewis acids. (b) PL spectra of compound **2** under different acid conditions in dichloromethane. (c) EL spectra of devices with compound **2** doped with CSA at different concentrations. Figure 3a was reprinted with permission from [26], Copyright 2002 American Chemical Society. This content is not subject to CC BY 4.0. Figure 3b and 3c were reproduced from [27] with permission from The Royal Society of Chemistry. This content is not subject to CC BY 4.0.

In 2002, Monkman reported the addition of camphor sulfonic acid (CSA) to the fluorescent polymer poly{2,5-pyridylene-*co*-1,4-[2,5-bis(2-ethylhexyloxy)]phenylene} (compound **1** in Figure 2) containing pyridine groups led to the protonation effect [26]. CSA has strong acidity and low volatility, which is feasible to be bound with pyridine groups. As shown in Figure 3a, the protonation by CSA resulted in a significant red-shift in the photoluminescence (PL) spectrum, which was similar to the cases caused by other Lewis acids such as methanesulfonic acid (MSA) and dichloracetic acid (DCA). Wang et al. used HCl, TFA, and BBr_3_ as dopants which were respectively added to the donor–acceptor–donor (D–A–D) molecule 2,5-bis((*N*,*N*-diphenylamino)phenyl)thiazolothiazole (compound **2** in Figure 2) containing thiazolothiazole units. As shown in Figure 3b, four different colors ranged from green, yellow, red and NIR regions, i.e., a dramatic wavelength shift of 215 nm [27]. Light-emitting devices were fabricated by adding different concentrations of CSA into the fluorescent compound and a wide range of color tunability was observed in the EL spectra (see Figure 3c).

In 2009, Welch et al. employed the Lewis acid B(C_6_F_5_)_3_ (BCF) to bind to nitrogen atoms at the basic site a of π-conjugated polymer, providing a simple strategy to regulate the optical properties of the A–D–A chromophore with charge transfer excited state properties [28]. In 2019, Wang et al. constructed a novel exciplex system by using the Lewis acids B(C_6_F_5_)_3_ and B(C_6_H_5_)_3_ as electron acceptors, respectively [29]. B(C_6_F_5_)_3_ displays high chemical stability and Lewis acidity [30]. Moreover, its good solubility endows the possibility to form Lewis acid–base adducts in films by solution processing. The strong electron attraction of the fluorine substituents on the benzene rings of B(C_6_F_5_)_3_ is responsible for its stronger Lewis acidity compared to B(C_6_H_5_)_3_, and reacted efficiently with the basic fluorescent materials.

**Figure 4:**
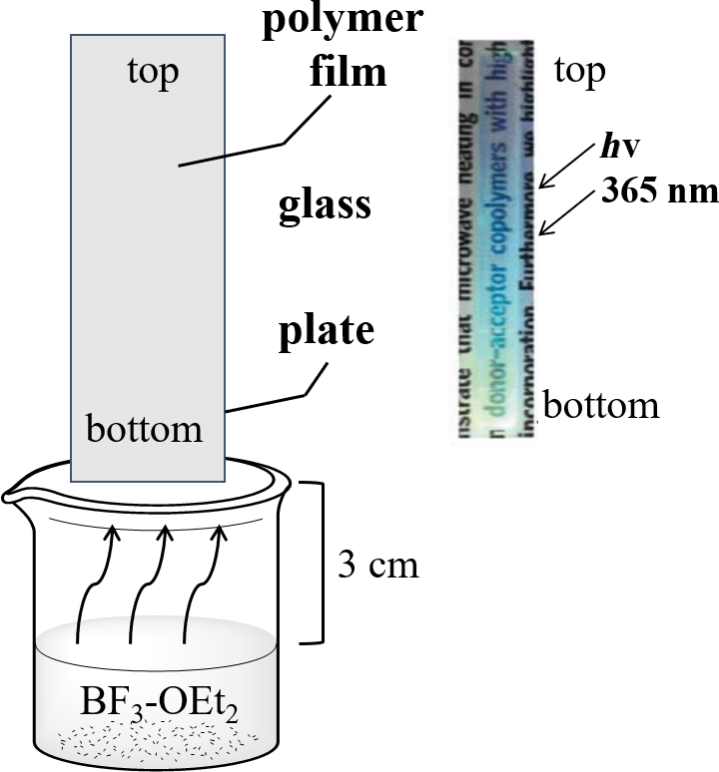
Schematic diagram of a BF_3_·OEt_2_ vapor-treated device and the macroscopic gradation emissive pattern of polymer films on a glass plate after treatment and excited by 365 nm UV light. Figure 4 was reproduced from [31] with permission from The Royal Society of Chemistry. This content is not subject to CC BY 4.0.

In 2011, Hayashi investigated the modification of pyridyl-conjugated polymer films with the Lewis acid BF_3_ [31]. Through repeated acid–base treatment, the polymer film can achieve reversible color changing. Due to the poor solubility, the doped polymer film was simply prepared by BF_3_ vapor treatment. The schematic diagram is shown in Figure 4. It is clear that the film achieved a gradient of colors from top to bottom under 365 nm UV light, which confirmed that the emission was sensitive to BF_3_ concentration. Yang et al. used also TFA to shape the fluorescence emission based on the protonation effect between the dissociated H^+^ and the fluorescent material [32].

Lin et al. used the Lewis acids B(C_6_F_5_)_3_ and AlCl_3_ to regulate the optoelectronic properties of a fluorene-based copolymer with an sp^2^ nitrogen heteroatom via supramolecular coordination [33]. The PL emission in solution showed an obvious red-shifted profile. The polymer LED with different molar equivalents of Lewis acids was investigated. The EL peak wavelength was gradually red-shifted with increasing the concentration of the Lewis acid, changing from 440 nm to 520 nm. In order to further explore the doping mechanism of Lewis acid on organic semiconductors, Yurash et al. found that B(C_6_F_5_)_3_ possessed the best doping effect and thus increased the conductivity, compared with BF_3_, BBr_3_, and AlCl_3_, respectively, mixed in the low bandgap conjugated polymer materials. This is ascribed to the formation of Lewis acid–base adducts [34].

### Fluorescent materials as electron donors

**Figure 5:**
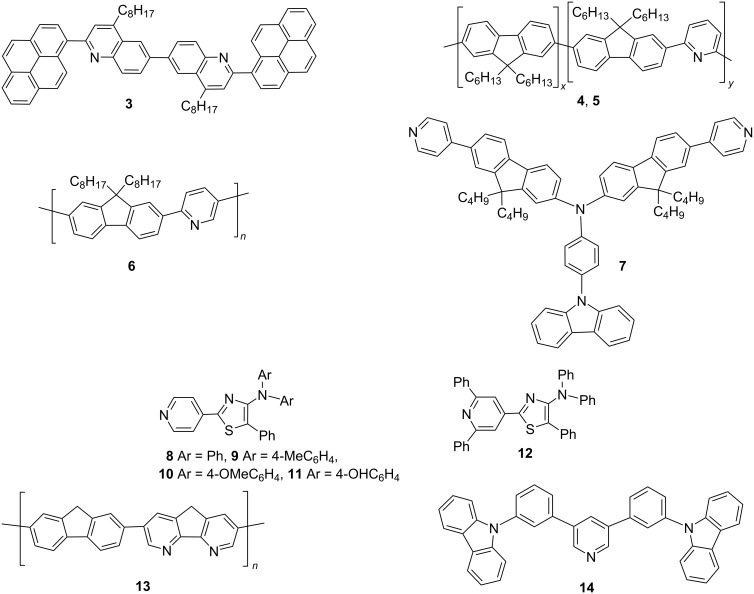
Chemical structures of Lewis basic fluorescent compounds **3–14**.

Hancock et al. compared the PL and EL spectra of the π-conjugated heterocyclic oligomer 6,6’-bis(2-(1-pyrenyl)-4-octylquinoline) (BPYOQ, compound **3** in Figure 5), which could be tuned in the whole visible range through the complex reaction with CSA [35]. This is supposed to be the first EL example of the protonated organic semiconductor. Compound **3** is an aromatic end-capped oligoquinoline, with both quinoline and pyridine as N-containing heterocycles rich in electrons, which are the key structural factors leading to acid discoloration. At the same time, Kappaun et al. synthesized a series of conjugated alternating and statistical copolymers (poly[2,7-(9,9-dihexylfluorenyl)-*alt*-(2,6-pyridinyl)]) (compound **4** in Figure 5) and (poly[2,7-(9,9-dihexylfluorenyl)-*stat*-(2,6-pyridinyl)]) (compound **5** in Figure 5) with pyrene and pyridine units [36]. The pyridine groups in the conjugated polymer contain basic sites presumably induced by nitrogen atoms, where protonation occurred.

**Figure 6:**
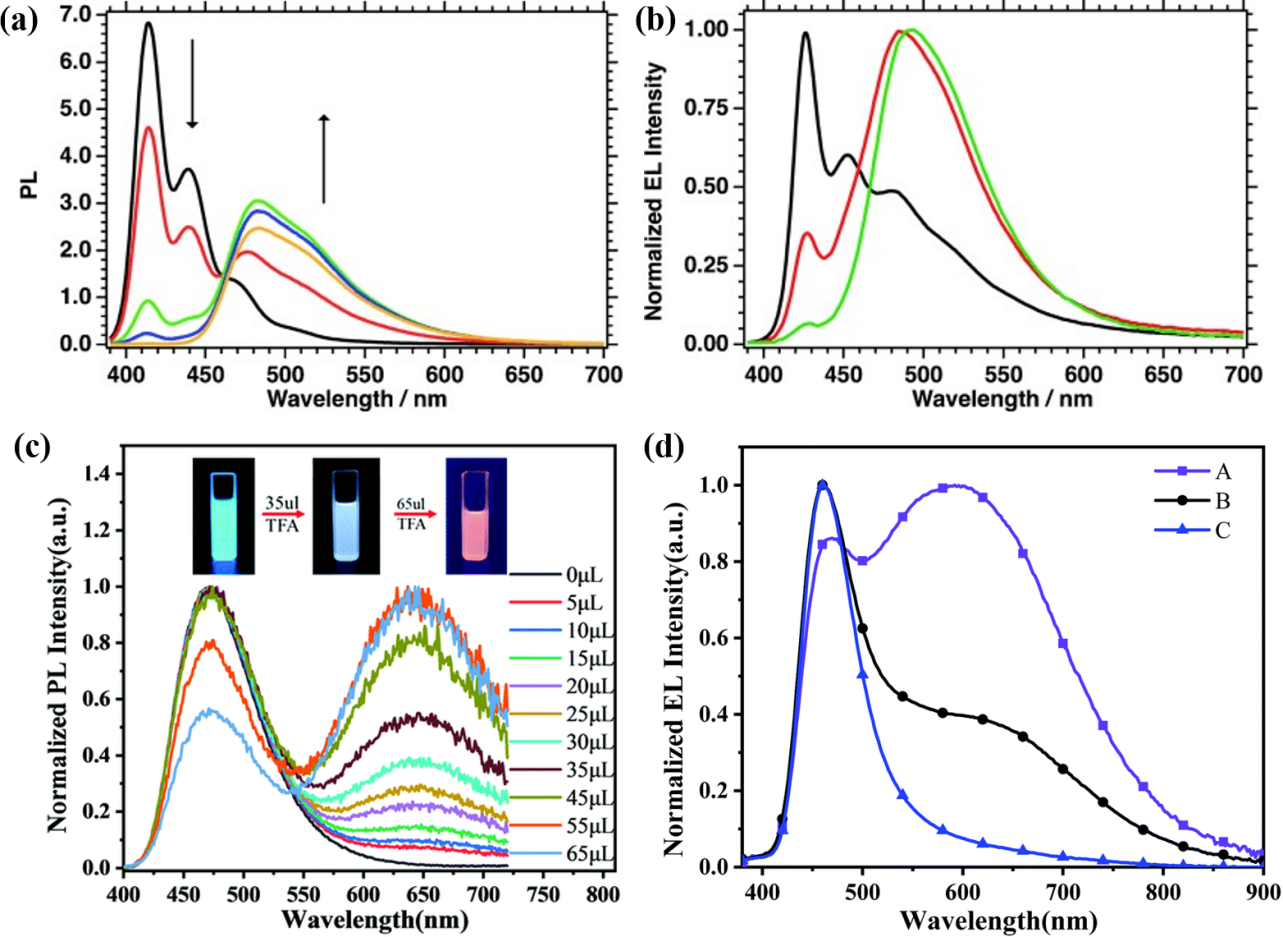
(a) PL spectra of compound **6** in toluene after addition of 0.0 (black line), 0.1 (red line), 0.3 (green line), 0.7 (blue line), 1.3 mol equiv (orange line) B(C_6_F_5_)_3_. (b) EL spectra of the device with compound **6** at a constant current density of 111 mA cm^−2^ for 0.00 (black line), 0.01 (red line), and 0.02 mol equiv (green) B(C_6_F_5_)_3_. (c) PL spectra of compound **7** in solution containing different amounts of TFA under irradiation of UV light. (d) EL spectra of devices with different ratios of compound **7** and TFA; device A, compound **7**/TFA 50:1 (v/v); device B, compound **7**/TFA 5000:1 (v/v); device C, neat film of compound **7**. Figure 6a and 6b were reproduced from [37], P. Zalar et al., “Color Tuning in Polymer Light-Emitting Diodes with Lewis Acids”, Angew. Chem., Int. Ed., with permission from John Wiley and Sons. Copyright © 2012 WILEY-VCH Verlag GmbH & Co. KGaA, Weinheim. This content is not subject to CC BY 4.0. Figure 6c and 6d were reproduced from [32] with permission from The Royal Society of Chemistry. This content is not subject to CC BY 4.0.

In 2012, Zalar et al. synthesized the conjugated polymer F8Py (compound **6** in Figure 5), in which the incorporation of the pyridine co-monomer provides a lone pair of electrons for binding Lewis acids [37]. The formation of acid–base adducts accurately regulated the band gap of the luminescent polymer. The PL spectra in solution showed the evident red-shift upon mixing the polymer with the Lewis acid (Figure 6a). This property was also successfully demonstrated in OLEDs to modify the electroluminescence (EL) characteristics (Figure 6b).

In 2020, Yang et al. designed and synthesized a blue fluorescent material CzPA-F-PD (compound **7** in Figure 5), which consisted of the twisted A–π–D–π–A structure with *N*-(4-aminophenyl)carbazole (CzPA) as electron donor unit, pyridine as electron acceptor unit, and 9,9-dioctylfluorene (F) as π-conjugated linker [32]. Compound **7** showed remarkable dual-fluorescence properties when mixed with a very small amount of trifluoroacetic acid (TFA). As shown in Figure 6c, the PL spectra in solution were dominated by the amount of TFA. At the appropriate ratio, the solution-processed device with compound **7** as single emission layer generated broadband white light emission under EL process (see Figure 6d).

**Figure 7:**
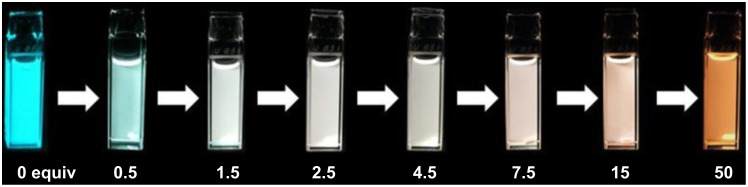
Photos of a solution of compound **12** and B(C_6_F_5_)_3_ at different ratios in toluene under a 365 nm UV lamp. Figure 7 was reproduced from [38] (© 2016 K. Yamaguchi et al., published by Wiley-VCH Verlag GmbH & Co. KGaA, distributed under the terms of the Creative Commons Attribution-NonCommercial 4.0 International License, https://creativecommons.org/licenses/by-nc/4.0/). This content is not subject to CC BY 4.0.

In 2016, Yamaguchi et al. designed and synthesized a series of 5-*N*-arylaminothiazoles with 4-pyridyl groups at the 2-position (compounds **8–12** in Figure 5), which behaved as strong Lewis basic sites [38]. After adding BCF to compound **12**, a new emission peak was generated in the orange-red region, accompanied with a decrease of the original blue emission, as shown in Figure 7. The PL emission changed from blue to orange. This phenomenon was also reproducible by adding other Lewis acids, such as BCl_3_ and AlCl_3_. Interestingly, white light emission was achievable by adjusting the ratio of B(C_6_F_5_)_3_. Regarding the materials developed by Lin et al., supramolecular coordination of PF8-co-DAF8 (**13**, Figure 5) with Lewis acids played an important role. They selected the more rigid 4,5-diazafluorene (DAF) with nitrogen atoms inserted at the 4 and 5-positons of the fluorene moiety [33]. The heteroatomic fluorene showed enhanced planarity of the molecule. The coordination tended to be more efficient if a stronger Lewis acid was employed.

The bipolar host material 35DCzPPy (**14**, Figure 5) was initially synthesized by Kido’s group [39]. It combines two carbazole electron donors with high triplet energy and a pyridine electron acceptor with high electron affinity. Later in 2020, Wang’s group employed this host material, respectively mixed with two Lewis acids, namely BCF and B(C_6_H_5_)_3_, to construct highly luminescent exciplexes [29]. The PL spectra of the new emission system showed an obvious red-shift through intermolecular charge transfer. Compared with B(C_6_H_5_)_3_, the exciplex system constructed by BCF exhibited a more pronounced red-shift in the PL spectra and unexpectedly improved EL properties.

The fluorescent materials, which can easily interact with Lewis acids and simultaneously exhibit significant chemical and photophysical changes, have some common structural characteristics. For instance, heterocyclic units containing a nitrogen atom such as pyridine and thiazole, are one of the key structural features either in small molecules or polymers. Thus, the introduction of nitrogen with lone pairs of electrons in fluorescent materials, makes them have a good affinity for Lewis acids. In other words, these fluorescent materials contain Lewis basic sites for the formation of Lewis acid–base pairs. According to this principle, it can be inferred that analogous materials containing basic nitrogen atoms tend to interact with the Lewis acids discussed in this review and thus lead to a significant shift of their optoelectronic properties. It has been confirmed that organic molecules containing pyrimidine, pyrazine, and indole groups display similar interactions upon the addition of Lewis acids [40–42].

### Lewis acid–base interaction mechanisms

#### Chemical essence of Lewis acid–base interaction

**Figure 8:**
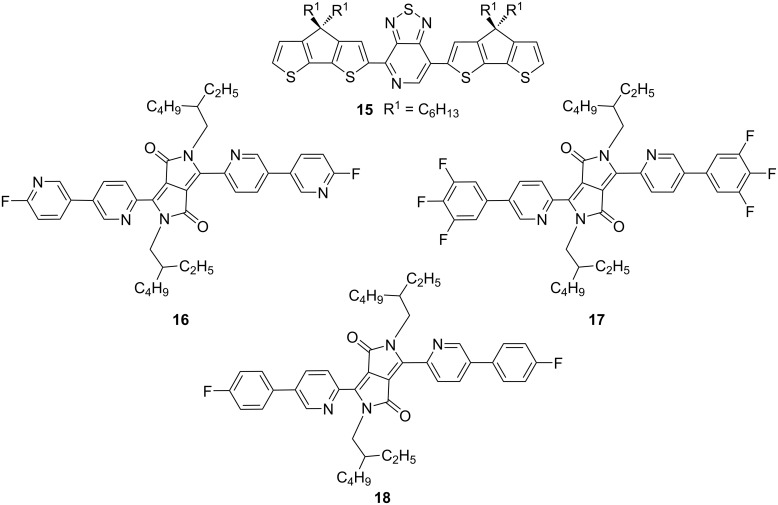
Structure of small molecule **15** containing pyridine and thiazole groups reported by Bazan et al. and pyridine groups-containing diketopyrrolopyrroles (DPP) **16**–**18** investigated by Huang et al.

**Figure 9:**
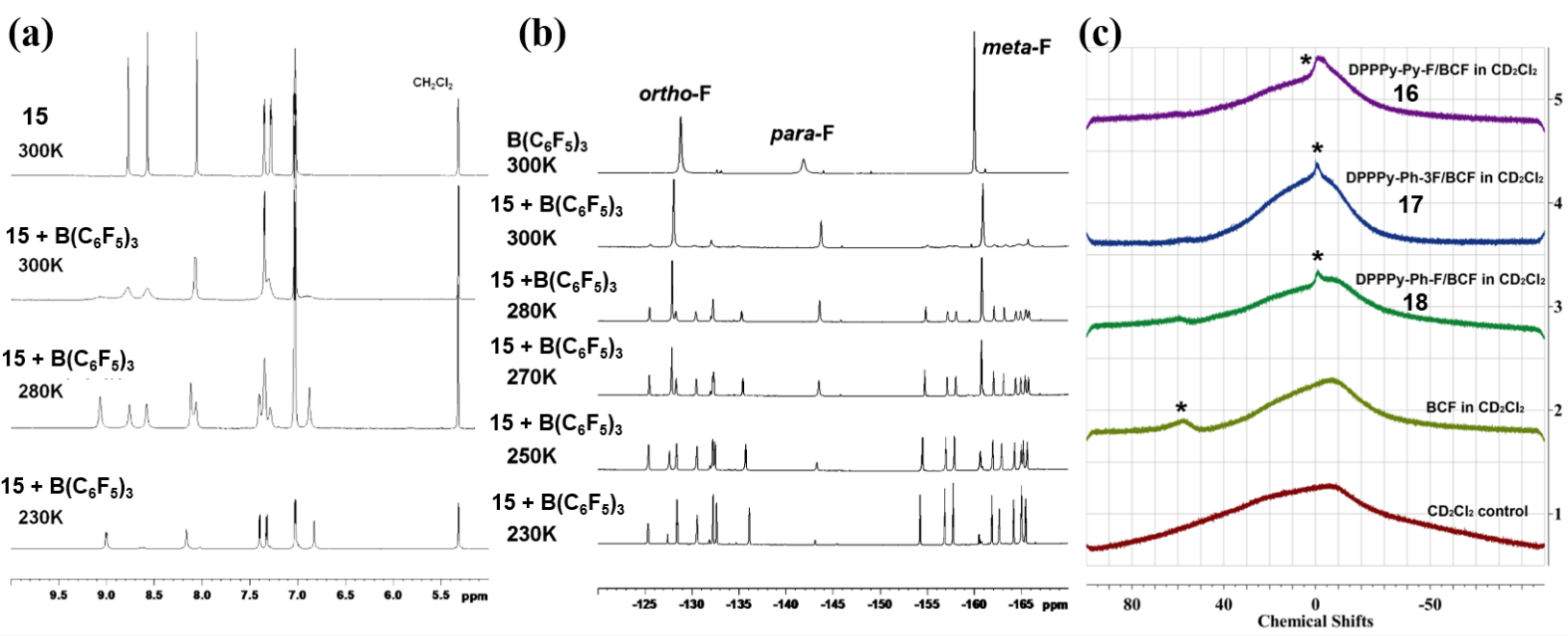
(a) ^1^H NMR spectra in the aromatic region and (b) ^19^F NMR spectra of compound **15** (top) and the mixture with 1 equivalent B(C_6_F_5_)_3_ at different temperatures from 300 to 230 K. (c) ^11^B NMR spectra of B(C_6_F_5_)_3_, DPPPy-Py-F (compound **16**)/B(C_6_F_5_)_3_, DPPPy-Ph-3F (compound **17**)/B(C_6_F_5_)_3_, and DPPPy-Ph-F (compound **18**)/B(C_6_F_5_)_3_ in CD_2_Cl_2_, respectively. Figure 9a and 9b were reprinted with permission from [43], Copyright 2011 American Chemical Society. This content is not subject to CC BY 4.0. Figure 9c was reprinted from [44], Dyes and Pigments, vol. 153, by J. Huang; Y. Li, Y. Wang; H. Meng; D. Yan; B. Jiang; Z. Wei; C. Zhan, “A Lewis acid-base chemistry approach towards narrow bandgap dye molecules”, pages 1–9, Copyright (2018), with permission from Elsevier. This content is not subject to CC BY 4.0.

All the above discussed fluorescent materials share the common characteristics of Lewis basicity. Therefore, the changes in band gaps and colors of the donor materials is essentially attributed to a Lewis acid–base complexation reaction. In order to clarify the coordination reaction of nitrogen atoms, Bazan’s group designed a conjugated polymer containing pyridine and thiazole groups and small molecule **15** (Figure 8) and compared the ^1^H NMR spectra and ^19^F NMR spectra after the addition of 1 equivalent B(C_6_F_5_)_3_ at various temperatures from 230 to 300 K (see Figure 9) [43].

As shown in Figure 9a, when the temperature reached 280 K, the aromatic resonances became intense, implying the appearance of a new species, which was assigned to the Lewis acid–base adduct. Fifteen new resonance peaks were also observed in the ^19^F NMR spectrum (see Figure 9b), which were different from the same chemical environment of fluorine atoms in the original B(C_6_F_5_)_3_. To further explore the interaction of the Lewis acid–base pairs, Huang et al. added B(C_6_F_5_)_3_ to pyridine group-capped diketopyrrolopyrrole (DPP) molecules, i.e., DPPPy-Py-F (**16**), DPPPy-Ph-3F (**17**), and DPPPy-Ph-F (**18**, Figure 8), and determined the ^11^B NMR spectra (Figure 9c) [44]. When coordinated with nitrogen atoms, the resonance peak shifted slightly from ca. −10 to 0 ppm, which suggested the interaction between boron and nitrogen atoms.

Wang’s group studied the interaction of compound **14** respectively with B(C_6_F_5_)_3_ and B(C_6_H_5_)_3_ by X-ray photoelectron spectroscopy (XPS) [29]. The B(1s) signal showed peaks at 190.61 and 191.08 eV, respectively. This is close to the reported characteristic B–N binding energy (190.5 eV) in B–N crystals. Despite the weak signals of boron in these two Lewis acids, it was assumed that compound **14** formed a B–N coordination bond when doped with B(C_6_F_5_)_3_ and B(C_6_H_5_)_3_, respectively.

### Luminescent mechanisms

**Figure 10:**
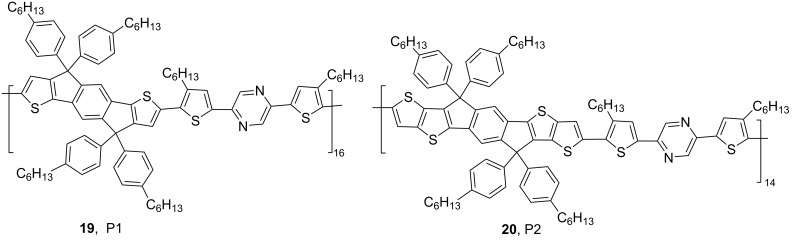
Pyrazine-containing polymers **19** and **20** investigated by Li et al.

**Figure 11:**
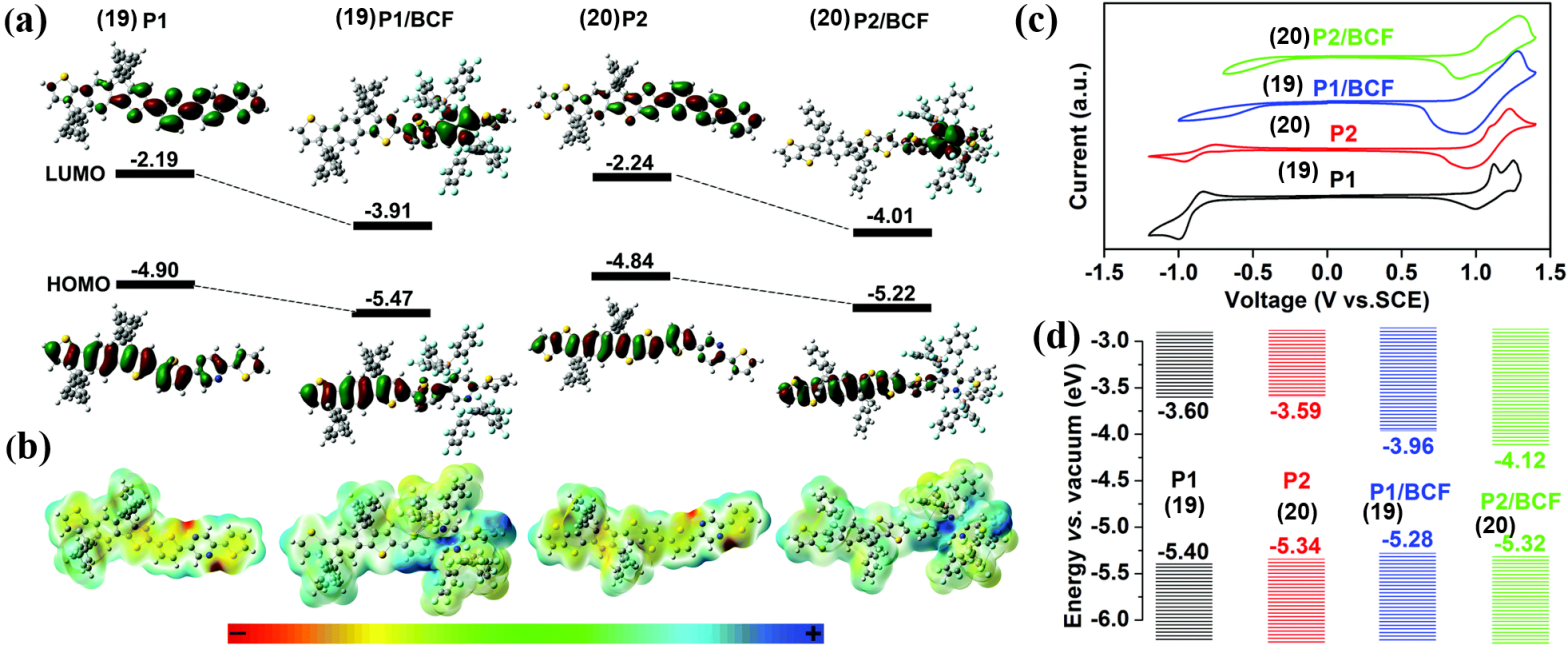
(a) HOMO/LUMO orbitals and energy levels (unit: eV) and (b) electrostatic potential surface (EPS) maps calculated by Gaussian 09 at the B3LYP/6-31G(d,p) level of theory. (c) Cyclic voltammetry curves of the four compounds and HOMO/LUMO energy level diagram and (d) estimated from the CV tests. Figure 11a–d were reproduced from [45] with permission from The Royal Society of Chemistry. This content is not subject to CC BY 4.0.

In view of the phenomenon that Lewis acid–base coordination contributes to a decrease of the band gap and bathochromic shifts of absorption and emission, it is essential to explore the mechanisms. Welch et al. supposed that the strong electrophilic Lewis acid triggers charge transfer with nitrogen-containing heterocycles containing lone-pair electrons. Consequently, it reduces the electron density of the π-conjugated system and the characteristics of the excited states, accounting for the decrease of band gap [27,37,43]. In 2018, Li et al. used density functional theory (DFT) to investigate the energy levels of polymers **19** (P1) and **20** (P2, Figure 10) containing pyrazine groups before and after the addition of B(C_6_F_5_)_3_ (see Figure 11a) [45]. Considering the electrostatic potential surface (EPS) maps (see Figure 11b) of the pyrazine-containing polymers before and after B(C_6_F_5_)_3_ coordination, it is likely that B(C_6_F_5_)_3_ sacrificed the electron density of the polymer skeleton and turned it from an electron-rich to an electron-deficient species. This was assumed to be the reason for the decrease of the band gap. Meanwhile, the LUMO levels estimated from electrochemistry experiments (see Figure 11c and 11d) were also depressed from −3.60 eV (compound **19**) to −3.96 eV (compound **19**/B(C_6_F_5_)_3_) and from −3.59 eV (compound **20**) to −4.12 eV (compound **20**/B(C_6_F_5_)_3_), which were consistent with the theoretical calculation results.

Yang and co-workers compared the energy level distributions of the HOMO and LUMO of CzPA-F-PD (compound **7** in Figure 5) before and after protonation, which were diverse [32]. The cyclic voltammogram (CV) curves of CzPA-F-PD and CzPA-F-PD-H^+^ showed that the energy levels of both the HOMO and LUMO of CzPA-F-PD-H^+^ decreased relative to those of CzPA-F-PD, and the LUMO level decreased more significantly. According to the theoretical calculation results, the HOMO and LUMO distributions of CzPA-F-PD-H^+^ were more spatially separated, the charge transfer characteristics of the excited states turned to be stronger, and the localized excited states characteristics was reduced. The energy level gap between S_1_ and T_1_ (Δ*E*_ST_) of CzPA-F-PD-H^+^ was 0.16 eV, which is significantly lower than the 0.39 eV of CzPA-F-PD [32].

**Figure 12:**
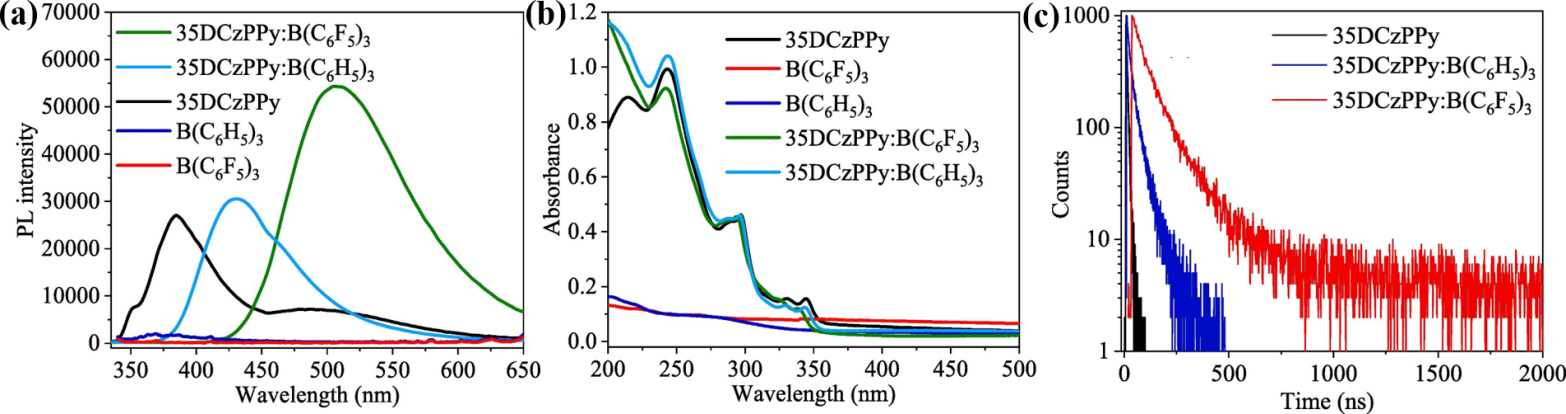
(a) UV–vis absorbance and (b) PL spectra (excited by 330 nm) for 35DCzPPy (compound **14**), B(C_6_F_5_)_3_, B(C_6_H_5_)_3_, 35DCzPPy:B(C_6_F_5_)_3_ (1:1), and 35DCzPPy:B(C_6_H_5_)_3_ (1:1) in films. (c) Fluorescence decay curves for the solid films of 35DCzPPy, 35DCzPPy:B(C_6_H_5_)_3_, and 35DCzPPy:B(C_6_F_5_)_3_ recorded at photoluminescence maxima (385, 435, and 509 nm) at room temperature. Figure 12 was reprinted from [29], Chemical Engineering Journal, vol. 380, by M. Zhang; G. Xie; Q. Xue; H. Wang, “Electroluminescence of intra-molecular exciplexes based on novel Lewis acid borane acceptors and a high triplet level donor”, article no. 122527, Copyright (2020), with permission from Elsevier. This content is not subject to CC BY 4.0.

The formation of exciplexes, e.g., with the donor-like 35DCzPPy (compound **14** in Figure 5) and acceptor-like Lewis acids, effectively reduces the energy gap between S_1_ and S_0_ and thus leads to a red-shift of emission (Figure 12a), as claimed by Xie and Wang’s group [29]. The absorption of both 35DCzPPy:B(C_6_F_5_)_3_ and 35DCzPPy:B(C_6_H_5_)_3_ were nearly identical to that of their constituting materials, which suggested that there existed no new ground-state in the exciplex films (Figure 12b). The reduction of the LUMO energy level would correlate closely with the protonation effect on the pyridine unit of the donor. More importantly, delayed fluorescence profiles of the exciplexes were detected (see Figure 12c, τ_1_ = 57.07 ns and τ_2_ = 158.20 ns), which proved the possibility to harvest triplet excitons based on Lewis acid–base adducts. Therefore, the OLED using 35DCzPPy:B(C_6_F_5_)_3_ as the emitting layer exhibited a maximum external quantum efficiency of ≈6.2%, surpassing the upper limit (ca. 5%) of the conventional fluorescence devices.

### Strength of Lewis acid–base interactions

**Figure 13:**
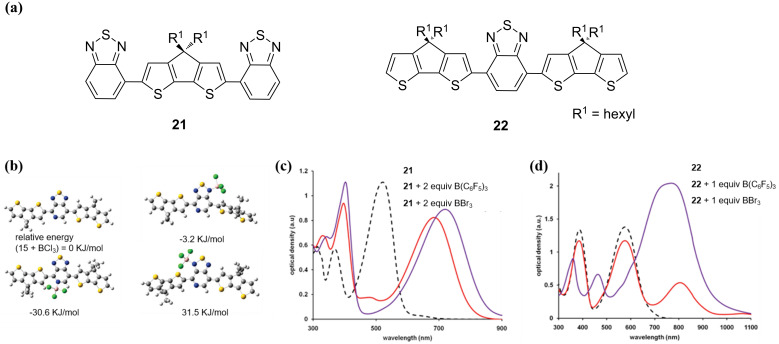
(a) Schematic diagram of the low-band gap materials **21** and **22**. (b) Ground state geometry optimizations of compound **15** and its corresponding adducts with BCl_3_. The optimized structures were calculated using DFT at the B3LYP/6-31G (d,p) level of theory. UV–vis–NIR absorption spectra of (c) compound **21** and (d) compound **22** before and after adding the Lewis acids B(C_6_F_5_)_3_ and BBr_3_ in *o*-DCB solution, respectively. Figure 13b–d were reprinted with permission from [43], Copyright 2011 American Chemical Society. This content is not subject to CC BY 4.0.

The energy levels of Lewis acid–base adducts are sensitive to the strength of the Lewis acids and bases. In 2002, Monkman et al. found that the degree of the spectral red-shift of protonated conjugated polymers depended greatly on the strength of the Lewis acid (Figure 3a) [26]. Wang et al. modulated the electron-accepting strength of intramolecular charge transfer molecules by using different acids and obtained four distinctly different solid-state emission colors of green (524 nm), yellow (576 nm), red (640 nm), and NIR (739 nm) (Figure 3b) [27]. The stronger Lewis acidity resulted in a stronger emission and bathochromic shift when comparing the effects of BCF and B(C_6_H_5_)_3_ on the optoelectronic properties of the organic UV fluorescent material 35DCzPPy (**14**, Figure 5) [29,39]. As illustrated in Figure 12a, BCF can narrow down the bandgap of the exciplex because of the stronger electrophilicity of the fluorine atoms. Similarly, Yamaguchi et al. used molecular modifications to introduce stronger electron donors to luminescent molecules and obtained stronger spectral changes [38]. This demonstrates that stronger Lewis acids and Lewis bases will result in stronger charge transfer. Moreover, stronger electron donors or more accessible nitrogen-containing groups would interact easily via Lewis acid coordination. As shown in Figure 13b, the energy levels determined from the optimized structures of compounds **21** and **22** (Figure 13a) by DFT suggest that pyridine is a better binding site than thiophene [43].

The effect of steric hindrance on the Lewis acid–base binding should not be ignored. If there is large steric hindrance of the Lewis basic molecules, it will hinder the coordination with a Lewis acid. For example, Bazan’s group investigated the analogous compounds **21** and **22** shown in Figure 13a, featuring the same nitrogen heterocycles but with different steric hindrances. Subsequently, the ability of their coordination with B(C_6_F_5_)_3_ and BBr_3_ was compared, respectively [43]. As displayed in Figure 13c and 13d, the UV–vis–NIR absorption spectra manifested that the larger steric hindrance interrupted the binding of BCF more effectively than that of BBr_3_.

## Conclusion

For fluorescent materials containing nitrogen atoms with Lewis basic nature, it is easily found that the addition of suitable Lewis acids can lead to a dramatic red-shift in the absorption and emission of the mixtures. The electrophilic Lewis acid as electron acceptor frequently reacts with the nitrogen-containing heterocyclic conjugated molecules, ascribed to the charge redistributions of the molecules. This governs their optoelectronic properties and most likely rouses the non-radiative triplet excitons of reverse intersystem crossing.

Lewis acid–base chemistry provides a simple and effective way to finely regulate the optoelectronic properties of fluorescent materials, avoiding the complicated molecular synthesis. Lewis acid–base interactions found some promising applications in band gap engineering, photoluminescence, and electroluminescence. The in-depth study of the mechanisms of this phenomenon could inspire the innovation in cutting-edge researches beyond organic light-emitting diodes [29,32], e.g., organic thin-film transistors [45–46], organic photovoltaics [47], and chemical sensing [48].
